# 1-Allyl-3-chloro-5-nitro-1*H*-indazole

**DOI:** 10.1107/S1600536813021995

**Published:** 2013-08-10

**Authors:** Hakima Chicha, El Mostapha Rakib, Domenico Spinelli, Mohamed Saadi, Lahcen El Ammari

**Affiliations:** a Laboratoire de Chimie Organique et Analytique, Université Sultan Moulay Slimane, Faculté des Sciences et Techniques, Béni-Mellal, BP 523, Morocco; b Dipartimento di Chimica ’G. Ciamician’, Università degli Studi di Bologna, Via Selmi 2, I-40126 Bologna, Italy; c Laboratoire de Chimie du Solide Appliquée, Faculté des Sciences, Université Mohammed V-Agdal, Avenue Ibn Battouta, BP. 1014, Rabat, Morocco

## Abstract

In the title compound, C_10_H_8_ClN_3_O_2_, the indazole ring system makes a dihedral angle of 7.9 (3)° with the plane through the nitro group. The allyl group is rotated out of the plane of the indazole ring system [N—N—C—C torsion angle = 104.28 (19)°]. In the crystal, mol­ecules are linked by C—H⋯O hydrogen bonds, forming zigzag chains propagating along the *b-*axis direction.

## Related literature

For the pharmacological activity of indazole derivatives, see: Baraldi *et al.* (2001 ▶); Rodgers *et al.* (1996 ▶); Li *et al.* (2003 ▶); Lin *et al.* (2008 ▶). For a similar compound, see: El Brahmi *et al.* (2012 ▶).
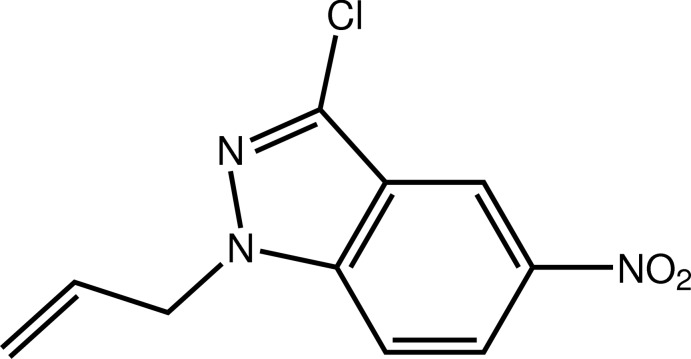


## Experimental

### 

#### Crystal data

C_10_H_8_ClN_3_O_2_
*M* *_r_* = 237.64
Monoclinic, 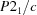

*a* = 13.3025 (6) Å
*b* = 11.2505 (5) Å
*c* = 7.3092 (3) Å
β = 91.343 (2)°
*V* = 1093.59 (8) Å^3^
*Z* = 4
Mo *K*α radiation
μ = 0.34 mm^−1^
*T* = 296 K
0.41 × 0.34 × 0.22 mm

#### Data collection

Bruker X8 APEX diffractometer
Absorption correction: multi-scan (*SADABS*: Sheldrick, 2008 ▶) *T*_min_ = 0.654, *T*_max_ = 0.747
14430 measured reflections
3069 independent reflections
1852 reflections with *I* > 2σ(*I*)
*R*_int_ = 0.046

#### Refinement

*R*[*F* ^2^ > 2σ(*F* ^2^)] = 0.047
*wR*(*F* ^2^) = 0.136
*S* = 1.02
3069 reflections
145 parameters
H-atom parameters constrained
Δρ_max_ = 0.27 e Å^−3^
Δρ_min_ = −0.33 e Å^−3^

### 

Data collection: *APEX2* (Bruker, 2009 ▶); cell refinement: *SAINT* (Bruker, 2009 ▶); data reduction: *SAINT*; program(s) used to solve structure: *SHELXS97* (Sheldrick, 2008 ▶); program(s) used to refine structure: *SHELXL97* (Sheldrick, 2008 ▶); molecular graphics: *ORTEP-3 for Windows* (Farrugia, 2012 ▶); software used to prepare material for publication: *PLATON* (Spek, 2009 ▶) and *publCIF* (Westrip, 2010 ▶).

## Supplementary Material

Crystal structure: contains datablock(s) I. DOI: 10.1107/S1600536813021995/bt6927sup1.cif

Structure factors: contains datablock(s) I. DOI: 10.1107/S1600536813021995/bt6927Isup2.hkl

Supplementary material file. DOI: 10.1107/S1600536813021995/bt6927Isup3.cml

Additional supplementary materials:  crystallographic information; 3D view; checkCIF report

## Figures and Tables

**Table 1:** Hydrogen-bond geometry (Å, °)

*D*—H⋯*A*	*D*—H	H⋯*A*	*D*⋯*A*	*D*—H⋯*A*
C6—H6⋯O2^i^	0.93	2.46	3.274 (2)	146

Symmetry code: (i) 
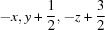
.

**Fig. 1.**
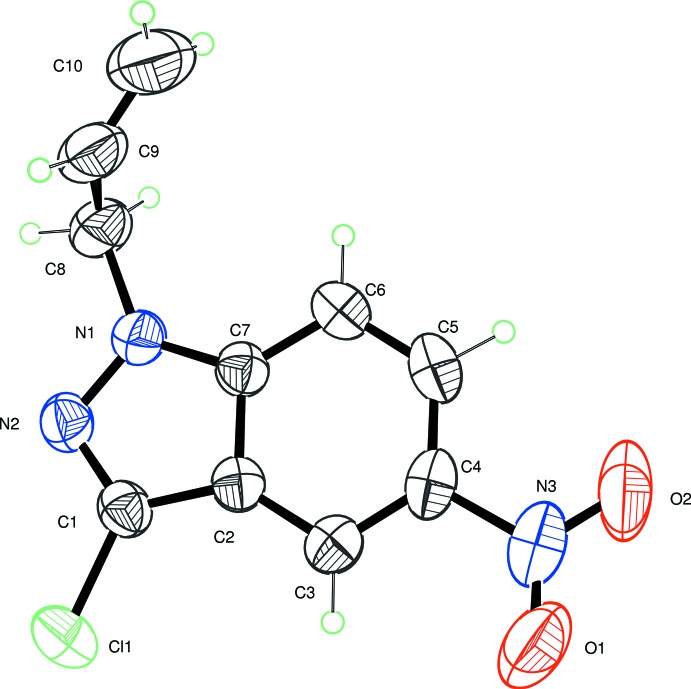
Molecular structure of the title compound with the atom-labelling scheme. Displacement ellipsoids are drawn at the 50% probability level. H atoms are represented as small circles.

**Fig. 2.**
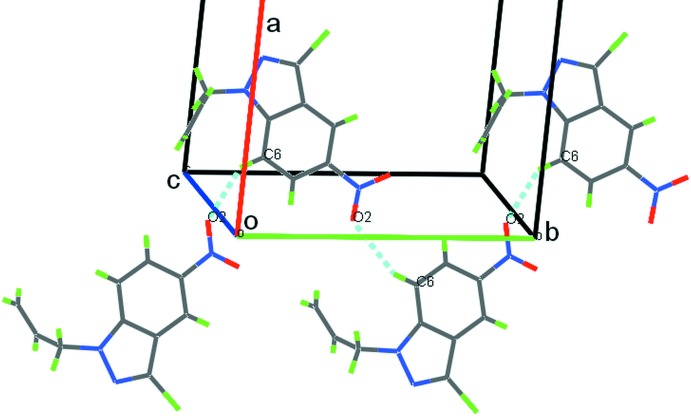
Partial crystal packing for the title compound showing C–H···O hydrogen bonds as dashed lines, showing a zigzag chain running along the b axis.

## supplementary crystallographic information

### 1. Comment

Indazole derivatives are an important class of heterocyclic pharmaceuticals because of their significant and broad spectrum of biological properties, including antitumor, anti-HIV, antimicrobial, anti-inflammatory, and contraceptive activities (Baraldi *et al.*, 2001; Rodgers *et al.*, 1996; Li *et al.*, 2003; Lin *et al.*, 2008). The present work is a contribution to the investigation of indazole derivatives (El Brahmi *et al.*, 2012).

In the molecule of 1-allyl-3-chloro-5-nitro-1*H*-indazole, the dihedral angle between the indazole system and the plan through the atoms forming the nitro group is of 7.9 (3)° and it is nearly perpendicular to the allyl group as indicated by the dihedral angle of 80.8 (3)°.

In the crystal, the molecules are interconnected by C–H···O hydrogen bonds forming zigzag chains running along the *b* axis as shown in Fig. 2 and Table 2.

### 2. Experimental

To a solution of 3-chloro-5-nitroindazole (6.13 mmol) in acetone (15 ml) was added potassium hydroxide (6.8 mmol). After 15 mn at 298 K, allyl bromide (12.26 mmol) was added dropwise. Upon disappearance of the starting material as indicated by TLC, the resulting mixture was evaporated. The crude material was dissolved with EtOAc (50 ml), washed with water and brine, dried over MgSO_4_ and the solvent was evaporated *in vacuo*. The resulting residue was purified by column chromatography (EtOAc/hexane 3/7). The title compound was recrystallized from ethanol.

### 3. Refinement

H atoms were located in a difference map and treated as riding with C–H = 0.97 Å, and C–H = 0.93 Å for methylene and aromatic H atoms, respectively. *U*(H) was set to 1.2*U*_iso_(C).

### Crystal data

**Table:**

C_10_H_8_ClN_3_O_2_	*F*(000) = 488
*M**_r_* = 237.64	*D*_x_ = 1.443 Mg m^−^^3^
Monoclinic, *P*2_1_/*c*	Mo *K*α radiation, λ = 0.71073 Å
Hall symbol: -P 2ybc	Cell parameters from 3069 reflections
*a* = 13.3025 (6) Å	θ = 2.4–29.6°
*b* = 11.2505 (5) Å	µ = 0.34 mm^−^^1^
*c* = 7.3092 (3) Å	*T* = 296 K
β = 91.343 (2)°	Block, colourless
*V* = 1093.59 (8) Å^3^	0.41 × 0.34 × 0.22 mm
*Z* = 4	

### Data collection

**Table:**

Bruker X8 APEX diffractometer	3069 independent reflections
Radiation source: fine-focus sealed tube	1852 reflections with *I* > 2σ(*I*)
Graphite monochromator	*R*_int_ = 0.046
φ and ω scans	θ_max_ = 29.6°, θ_min_ = 2.4°
Absorption correction: multi-scan (*SADABS*: Sheldrick, 2008)	*h* = −18→18
*T*_min_ = 0.654, *T*_max_ = 0.747	*k* = −10→15
14430 measured reflections	*l* = −9→10

### Refinement

**Table:**

Refinement on *F*^2^	Primary atom site location: structure-invariant direct methods
Least-squares matrix: full	Secondary atom site location: difference Fourier map
*R*[*F*^2^ > 2σ(*F*^2^)] = 0.047	Hydrogen site location: difference Fourier map
*wR*(*F*^2^) = 0.136	H-atom parameters constrained
*S* = 1.02	*w* = 1/[σ^2^(*F*_o_^2^) + (0.069*P*)^2^ + 0.0517*P*] where *P* = (*F*_o_^2^ + 2*F*_c_^2^)/3
3069 reflections	(Δ/σ)_max_ < 0.001
145 parameters	Δρ_max_ = 0.27 e Å^−^^3^
0 restraints	Δρ_min_ = −0.33 e Å^−^^3^

### Special details

**Table:**

Geometry. All e.s.d.'s (except the e.s.d. in the dihedral angle between two l.s. planes) are estimated using the full covariance matrix. The cell e.s.d.'s are taken into account individually in the estimation of e.s.d.'s in distances, angles and torsion angles; correlations between e.s.d.'s in cell parameters are only used when they are defined by crystal symmetry. An approximate (isotropic) treatment of cell e.s.d.'s is used for estimating e.s.d.'s involving l.s. planes.
Refinement. Refinement of *F*^2^ against all reflections. The weighted *R*-factor *wR* and goodness of fit *S* are based on *F*^2^, conventional *R*-factors *R* are based on *F*, with *F* set to zero for negative *F*^2^. The threshold expression of *F*^2^ > σ(*F*^2^) is used only for calculating *R*-factors(gt) *etc*. and is not relevant to the choice of reflections for refinement. *R*-factors based on *F*^2^ are statistically about twice as large as those based on *F*, and *R*- factors based on all data will be even larger.

### Fractional atomic coordinates and isotropic or equivalent isotropic displacement parameters (Å^2^)

**Table:**

	*x*	*y*	*z*	*U*_iso_*/*U*_eq_	
C1	0.39644 (11)	0.25338 (16)	1.0420 (2)	0.0451 (4)	
C2	0.29681 (11)	0.22952 (14)	0.97697 (19)	0.0415 (4)	
C3	0.24119 (12)	0.12846 (15)	0.9339 (2)	0.0462 (4)	
H3	0.2681	0.0525	0.9450	0.055*	
C4	0.14423 (13)	0.14758 (16)	0.8741 (2)	0.0517 (4)	
C5	0.10115 (12)	0.26115 (18)	0.8562 (2)	0.0549 (5)	
H5	0.0346	0.2685	0.8160	0.066*	
C6	0.15549 (13)	0.36043 (16)	0.8970 (2)	0.0508 (4)	
H6	0.1278	0.4360	0.8848	0.061*	
C7	0.25499 (12)	0.34380 (14)	0.9583 (2)	0.0428 (4)	
C8	0.32262 (15)	0.55248 (16)	1.0068 (3)	0.0596 (5)	
H8A	0.3794	0.5846	1.0763	0.071*	
H8B	0.2616	0.5785	1.0650	0.071*	
C9	0.32387 (17)	0.59986 (17)	0.8194 (3)	0.0666 (5)	
H9	0.3814	0.5866	0.7526	0.080*	
C10	0.2520 (2)	0.6582 (2)	0.7404 (5)	0.1088 (10)	
H10A	0.1931	0.6734	0.8025	0.131*	
H10B	0.2588	0.6852	0.6210	0.131*	
N1	0.32739 (10)	0.42252 (13)	1.01153 (19)	0.0492 (4)	
N2	0.41482 (10)	0.36715 (13)	1.06173 (19)	0.0503 (4)	
N3	0.08324 (14)	0.04395 (18)	0.8226 (2)	0.0720 (5)	
O1	0.12321 (14)	−0.05345 (16)	0.8167 (3)	0.0968 (6)	
O2	−0.00516 (13)	0.06089 (18)	0.7844 (3)	0.1118 (7)	
Cl1	0.48743 (3)	0.15046 (5)	1.08995 (7)	0.0677 (2)	

### Atomic displacement parameters (Å^2^)

**Table:**

	*U*^11^	*U*^22^	*U*^33^	*U*^12^	*U*^13^	*U*^23^
C1	0.0412 (8)	0.0522 (10)	0.0417 (8)	0.0014 (7)	−0.0009 (6)	0.0049 (7)
C2	0.0419 (8)	0.0462 (9)	0.0364 (7)	−0.0014 (7)	0.0006 (6)	0.0022 (7)
C3	0.0520 (10)	0.0463 (9)	0.0404 (8)	−0.0039 (7)	0.0034 (7)	0.0008 (7)
C4	0.0497 (10)	0.0619 (12)	0.0435 (8)	−0.0181 (8)	0.0021 (7)	−0.0055 (8)
C5	0.0388 (9)	0.0757 (13)	0.0500 (10)	−0.0016 (9)	−0.0047 (7)	−0.0016 (9)
C6	0.0458 (9)	0.0567 (11)	0.0498 (9)	0.0071 (8)	−0.0024 (7)	0.0017 (8)
C7	0.0428 (8)	0.0458 (9)	0.0398 (8)	−0.0012 (7)	−0.0008 (6)	0.0017 (7)
C8	0.0678 (12)	0.0427 (10)	0.0679 (12)	−0.0045 (8)	−0.0037 (9)	−0.0069 (8)
C9	0.0795 (14)	0.0424 (11)	0.0778 (13)	−0.0081 (10)	−0.0029 (11)	0.0029 (10)
C10	0.139 (3)	0.0582 (14)	0.127 (2)	−0.0137 (14)	−0.056 (2)	0.0176 (14)
N1	0.0485 (8)	0.0440 (8)	0.0548 (8)	−0.0036 (6)	−0.0071 (6)	0.0020 (6)
N2	0.0438 (8)	0.0544 (9)	0.0523 (8)	−0.0044 (6)	−0.0057 (6)	0.0045 (7)
N3	0.0667 (11)	0.0798 (13)	0.0694 (11)	−0.0279 (10)	0.0027 (9)	−0.0118 (10)
O1	0.0979 (12)	0.0673 (11)	0.1254 (15)	−0.0298 (10)	0.0049 (11)	−0.0242 (10)
O2	0.0639 (10)	0.1174 (15)	0.1528 (18)	−0.0334 (10)	−0.0243 (10)	−0.0240 (12)
Cl1	0.0516 (3)	0.0709 (4)	0.0803 (4)	0.0142 (2)	−0.0084 (2)	0.0080 (2)

### Geometric parameters (Å, º)

**Table:**

C1—N2	1.310 (2)	C7—N1	1.359 (2)
C1—C2	1.423 (2)	C8—N1	1.464 (2)
C1—Cl1	1.7056 (17)	C8—C9	1.470 (3)
C2—C3	1.389 (2)	C8—H8A	0.9700
C2—C7	1.406 (2)	C8—H8B	0.9700
C3—C4	1.369 (2)	C9—C10	1.286 (3)
C3—H3	0.9300	C9—H9	0.9300
C4—C5	1.405 (3)	C10—H10A	0.9300
C4—N3	1.465 (2)	C10—H10B	0.9300
C5—C6	1.360 (2)	N1—N2	1.3621 (19)
C5—H5	0.9300	N3—O2	1.217 (2)
C6—C7	1.400 (2)	N3—O1	1.219 (2)
C6—H6	0.9300		
			
N2—C1—C2	113.02 (14)	C6—C7—C2	121.48 (15)
N2—C1—Cl1	120.71 (12)	N1—C8—C9	112.52 (16)
C2—C1—Cl1	126.27 (14)	N1—C8—H8A	109.1
C3—C2—C7	121.26 (15)	C9—C8—H8A	109.1
C3—C2—C1	135.84 (16)	N1—C8—H8B	109.1
C7—C2—C1	102.90 (14)	C9—C8—H8B	109.1
C4—C3—C2	115.89 (16)	H8A—C8—H8B	107.8
C4—C3—H3	122.1	C10—C9—C8	125.4 (3)
C2—C3—H3	122.1	C10—C9—H9	117.3
C3—C4—C5	123.49 (16)	C8—C9—H9	117.3
C3—C4—N3	117.96 (18)	C9—C10—H10A	120.0
C5—C4—N3	118.54 (17)	C9—C10—H10B	120.0
C6—C5—C4	120.86 (16)	H10A—C10—H10B	120.0
C6—C5—H5	119.6	C7—N1—N2	111.97 (13)
C4—C5—H5	119.6	C7—N1—C8	127.90 (15)
C5—C6—C7	117.01 (16)	N2—N1—C8	119.96 (14)
C5—C6—H6	121.5	C1—N2—N1	105.13 (13)
C7—C6—H6	121.5	O2—N3—O1	123.54 (19)
N1—C7—C6	131.54 (16)	O2—N3—C4	117.4 (2)
N1—C7—C2	106.97 (14)	O1—N3—C4	119.02 (18)

### Hydrogen-bond geometry (Å, º)

**Table:**

*D*—H···*A*	*D*—H	H···*A*	*D*···*A*	*D*—H···*A*
C6—H6···O2^i^	0.93	2.46	3.274 (2)	146

Symmetry code: (i) −*x*, *y*+1/2, −*z*+3/2.
